# Density of nerve fibres and expression of substance P, NR2B‐receptors and nerve growth factor in healthy human masseter muscle: An immunohistochemical study

**DOI:** 10.1111/joor.13109

**Published:** 2020-10-21

**Authors:** Abdelrahman M. Alhilou, Akiko Shimada, Camilla I. Svensson, Malin Ernberg, Brian E. Cairns, Nikolaos Christidis

**Affiliations:** ^1^ Devision of Oral Diagnostics and Rehabilitation Department of Dental Medicine Karolinska Institutet Scandinavian Center for Orofacial Neurosciences (SCON) Huddinge Sweden; ^2^ Department of Restorative Dentistry College of Dentistry Umm Al‐Qura University Makkah al Mukarramah Saudi Arabia; ^3^ Department of Geriatric Dentistry Osaka Dental University Osaka Japan; ^4^ Department of Physiology and Pharmacology Center for Molecular Medicine Karolinska Institutet Stockholm Sweden; ^5^ Faculty of Pharmaceutical Sciences University of British Columbia Vancouver BC Canada

**Keywords:** expression, immunohistochemistry, muscle biopsy, nerve growth factor, NR2B, substance P

## Abstract

**Background:**

In skeletal muscle, free nerve endings are mostly located within the connective tissue. However, the distribution of sensory afferent fibres in healthy human masseter muscle tissues has not been studied.

**Objectives:**

Primarily to investigate human masseter muscle nerve fibre densities as well as expression of NR2B receptors, substance P (SP) and nerve growth factor (NGF), and secondarily to compare this between a) nerve fibres associated with myocytes and within connective tissue; b) sexes; and c) ages.

**Methods:**

Microbiopsies of the masseter muscle were obtained from 60 sex‐ and age‐matched healthy participants. Biopsy sections were analysed using immunohistochemistry and were visualised with a Leica TCS SPE confocal microscope. The Mann‐Whitney U test was used for statistical analyses.

**Results:**

The density of nerve fibres within connective tissue was significantly greater than in nerve fibres associated with myocytes (*P *< .001). Nerve fibres within connective tissue expressed SP alone or together with NR2B significantly more often than those associated with myocytes (*P *< .001). The frequency of nerve fibres, which expressed SP alone or in combination with NR2B or NGF, was significantly greater in women than in men (*P *< .050). Moreover, the co‐expression of the three markers together was inversely correlated with age in women (*P *< .002).

**Conclusions:**

There is a higher density and greater expression of sensory nerve fibres within the connective tissue than associated with myocytes in healthy human masseter muscle. This suggests that nerve fibres within connective tissue are more involved in nociception than nerve fibres associated with myocytes.

## 1 BACKGROUND

The masseter muscle is one of the skeletal muscles that plays an important role in mastication. Skeletal muscles are composed of muscle fibres or cells (myocytes), connective tissue, blood vessels and nerves. The mandibular division of the trigeminal nerve innervates the masseter muscle, carrying both motor and sensory nerve fibres.^1^, ^2^ The motor component is responsible for coordinating both isometric and isotonic contractions of the masseter muscle, while the sensory component provides information on the position, movement and condition of muscle tissue. Some non‐specialised, small diameter sensory afferent fibres (nociceptors) convey nerve impulses to the central nervous system that may result in the perception of muscle pain. These sensory afferent fibres are located throughout most of the tissues in the muscle.^3^ However, it has not been determined in the human masseter muscle whether these putative nociceptors have a greater density in connective tissue or in association with myocytes.

The study of ageing is affected by multiple confounding factors. However, any factor that has a direct or indirect influence on pain is considered important for both researchers and clinicians.^4^ Age can influence the functional, morphological and biochemical features of the peripheral nervous system (PNS).^5^ By the age of 60 years old, the density of peripheral nerve fibres is reported to be decreased,^6^, ^7^ with a greater reduction in the unmyelinated than myelinated fibres.^5^ Substance P (SP) is a neuropeptide expressed by small sensory afferent fibres, some of which are thought to convey nociceptive input from the muscle,^3^ and so a decrease in neuropeptide expression in nerve fibres could also reflect a reduction of nociceptive nerve fibre density. In animal biochemical studies, the content of neuropeptides such as SP has been reported to be reduced with age in the lumbar and thoracic dorsal root ganglion cells ^8^ as well as in the tooth pulp.^9^ In humans, a decrease in SP content has been reported with age in the primary afferent fibres of the skin.^10^ However, the association between age and the neurobiology of sensory afferent fibres in healthy masseter muscle tissues remains unclear.

Nerve growth factor (NGF) is a neurotrophin, which has important roles in neuronal survival, growth and apoptosis.^11^ Moreover, studies have demonstrated that it can modulate peripheral nociception and pain, especially in inflammatory conditions, through the activation of two different membrane‐bound receptors, the p75 receptor and the tyrosine kinase A (TrkA) receptor.^12^, ^13^, ^14^ Most of the studies on the effect of NGF on masseter muscle afferent fibres have investigated the expression of TrkA, N‐methyl‐D‐aspartate (NMDA) receptors and/or neuropeptides, such as SP and calcitonin gene‐related peptide (CGRP).^15^, ^16^ However, NGF expression by muscle nerve fibres itself is not well addressed in the literature.

Injection of NGF and glutamate into human masticatory muscles has been used experimentally to mimic symptoms of patients suffering from myofascial temporomandibular disorders (TMD). NGF injection induces local signs of mechanical allodynia and hyperalgesia that last for at least one week, with little or no ongoing pain.^17^, ^18^, ^19^ In contrast, glutamate injection evokes short duration, high‐intensity pain but mechanical sensitisation that lasts for only a few hours.^20^, ^21^ In both models, the magnitude of induced pain was reported to be greater in women than in men. For glutamate‐evoked muscle pain, this sex‐related difference could be due to an increased expression of NMDA receptor subtype 2B (NR2B) in women, because, in female rats, expression of this receptor is positively correlated with oestrogen levels.^22^ In NGF‐induced muscle pain, both the expression of SP and NR2B was increased in trigeminal ganglion neurons that innervate the masseter muscle of female rats.^16^ However, in healthy humans, it is unknown if the expression of NR2B or NGF by sensory neurons differs between sexes, or if the difference in expression occurs only as a consequence of a phenotypic modification of sensory nerve fibres induced by NGF or glutamate.

With this in mind and due to the limited knowledge about the sex, age and/or tissue differences in the neuro‐physiology/‐anatomy of the healthy human masseter muscle, this study aimed to characterise the presence of some molecular markers in the masseter muscle of healthy humans. Therefore, the primary aim was to investigate human masseter muscle nerve fibre densities as well as expression of SP, NR2B and NGF alone or in combination in the fibres; and secondarily to compare this between **a)** nerve fibres associated with myocytes and nerve fibres within connective tissue; **b)** sexes; and **c)** ages.

## 2 METHODS

### 2.1 Participants

Sixty healthy volunteers (30 women and 30 age‐matched men) with a mean ± standard deviation (SD) age of 28 (± 10) years participated in the study. The participants were recruited by one of the authors (AS), who had a position at the Aarhus University, Denmark, at the time of the study, by ads posted at Aarhus University and on an Internet page www.forsoegsperson.dk.

Inclusion criteria were age over 20 years and good general health. Exclusion criteria were facial pain or palpatory tenderness, systemic inflammatory diseases, neurological disorders, whiplash‐associated disorders, fibromyalgia, neuropathic pain, pregnancy, continuous use of medication except for birth control pills and the use of analgesic or anti‐inflammatory medication during the 24 hours preceding the biopsy. After receiving information about the study protocol, participants were screened for trial suitability at separate visits with a clinical examination performed according to the diagnostic criteria for TMD (DC/TMD).^23^ The DC/TMD clinical examination was added to ensure that the participants did not have any palpatory tenderness over the oro‐facial structures that could influence the results. The study followed the principles for medical research, according to the Declaration of Helsinki. Ethical approval was obtained from the region of Midtjylland, Denmark (August 27, 2015). Written and verbal information was received by the participants, and they gave their written consent prior to inclusion.

### 2.2 Collection of microbiopsies from the masseter muscle

**Figure 1:**
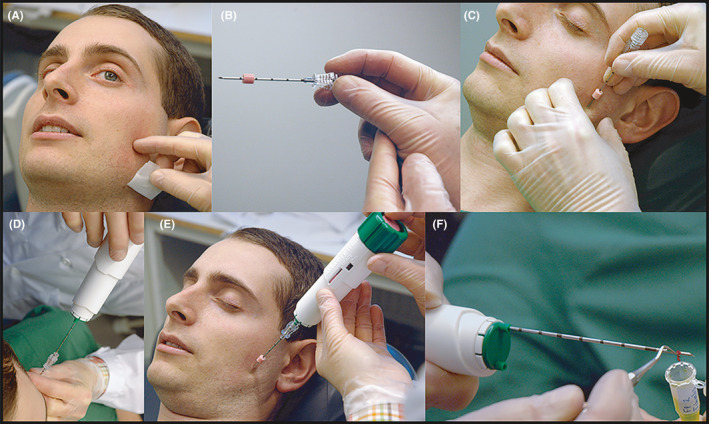
Illustration of the microbiopsy technique. A, The participant was asked to clench to allow the investigator identify the most prominent point of the masseter muscle by digital palpation. B, The Bard^®^TruGuide™ coaxial needle (BARD Norden, Helsingborg, Sweden) with its pink marker for the depth of insertion, that was used to guide the disposable Monopty^®^Bard^®^ biopsy instrument. C, The Bard^®^TruGuide^™^ coaxial needle was inserted to a depth of 10 mm. D, The biopsy instrument with a penetration depth of 11 mm and a diameter of 18G was inserted in the coaxial needle. E, By pressing the trigger button the biopsy instrument collected a piece of the muscle. F, The muscle section was removed from the biopsy instrument with a sterile, blunt probe and placed in 4% paraformaldehyde

A novel and unique microbiopsy method, developed by Christidis and co‐workers in 2014 for obtaining a sufficient amount of muscle tissue with only minor inconvenience for the participant, was used.^24^ Microbiopsies, with a mean weight of approximately 20 mg and a mean volume of approximately 13 mm^3^,^24^ were obtained by inserting a microbiopsy instrument through the skin and into the most prominent part of the masseter muscle. After 30 minutes of skin surface anaesthesia with a prefabricated anaesthetic patch (EMLA Patch^®^, 25mg lidocaine and 25 mg prilocaine, AstraZeneca, Södertälje, Sweden), a disposable coaxial biopsy needle (Bard^®^TruGuide™; BARD Norden, Helsingborg, Sweden) was inserted along the near long axis of the muscles until the fascia was penetrated to standardised depth of 10 mm. A disposable Monopty^®^Bard^®^ biopsy instrument (18G) was passed through the coaxial needle to a penetration depth of 11 mm and used to collect the masseter muscle biopsy (Figure 1). To ensure that an equivalent region was sampled across all participants, the guiding coaxial needle was inserted at an angle of 45 degrees, 1 cm below the zygomatic arch, to a depth of 1.0 cm. This allowed the muscle biopsy to be obtained from the most prominent part of masseter muscle for each subject.

### 2.3 Blinding of samples

In order to achieve a blinded analysis, all the microbiopsies were obtained and coded by one researcher (AS) not participating in the analysis. These codes were not revealed to the researcher (AA) who performed the analysis until all pictures were analysed.

### 2.4 Immunohistochemistry

The immunohistochemical analysis was conducted at the Faculty of Pharmaceutical Sciences, University of British Columbia, Vancouver, Canada, by AA and BEC. The microbiopsies were fixed with 4% paraformaldehyde at 4°C overnight. After that, they were rinsed in phosphate‐buffered saline (PBS), dehydrated, then frozen in a − 80 freezer until sectioned. The microbiopsies were placed in a plastic mould (Tissue‐Tek disposable vinyl specimen mould, 10mm * 10mm *5mm) and embedded in optimal cutting temperature (OCT) compound (Fisher Scientific, Ontario, Canada). Sections were sliced using a cryotome to a thickness of 10 μm and then mounted on glass slides and stored at 37°C overnight. The sections were incubated in normal donkey serum for 1 hour and then for 24 hours with primary antibodies against the specific axonal marker PGP 9.5 (1:250; anti‐PGP 9.5 antibody, ABCAM Inc, Cambridge, England; ab72911) and antibodies against the NR2B subunit (1:200; anti‐NMDAR2B antibody, ABCAM Inc, Cambridge, England; ab65783), SP (1:1000; anti‐SP antibody, ABCAM Inc, Cambridge, England; ab10353) and NGF (1:20; Human beta‐NGF Affinity Purified Polyclonal Ab, R&D Systems Inc, 614 McKinley PL NE Minneapolis, AF‐256‐NA). The specificity of the antibodies has been previously demonstrated.^16^ Sections were rinsed with PBS and incubated with fluorescent secondary antibodies (Alexa 488 donkey anti‐mouse, 1:700 for PGP 9.5, and Alexa Fluor 546 donkey anti‐rabbit, 1:700 for NR2B, and Alexa Fluor 633 donkey anti‐goat, 1:700 for NGF, Thermo Fisher, Burlington, ON, Canada. Alexa Fluor 405 donkey anti‐guinea pig, 1:700 for SP, Sigma‐Aldrich, MO, USA). A Leica TCS SPE Confocal Microscope (Leica microsystems, Wetzlar, Germany) was used to visualise the sections, and images for analysis were captured with a Leica scanner attached to the microscope. The omission of the primary antibodies did not result in specific staining of tested sections.

### 2.5 Data analysis

**Figure 2:**
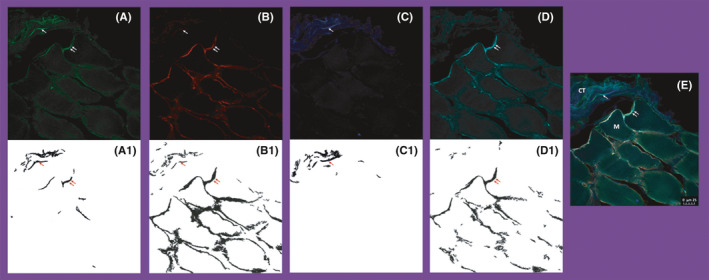
This figure is an example of high power (40 x) photomicrographs from a single section containing both myocytes and connective tissue, from one male participant. In part (E), the letter M demonstrates a myocyte while the letters CT demonstrates connective tissue. The presented pictures are showing fluorescent signalling of (A) PGP 9.5, (B) SP, (C) NR2B and (D) NGF. In (A1) the mask used for analysis of PGP 9.5 is shown. The mask for SP is shown in (B1), for NR2B in (C1), and for NGF in (D1). The single arrow in the masks indicates the colocalisation of the molecule with the nerve fibre within connective tissue, while double arrow indicates it associated with myocytes. Scale bar = 25 µm

Image and statistical analyses were conducted at the Division of Oral Diagnostics and Rehabilitation, Department of Dental Medicine, Karolinska Institutet, by AA, CS and NC. Pictures were taken of all positive fibres found, which created variations in the number of images obtained between participants. However, not all participants had myocytes and connective tissue together in their biopsies, which led to different numbers of subject samples (Table 1). By using the image processing and analysis program ImageJ (Image Processing and Analysis in Java; National Institutes of Health, USA), a mask for every image was created (Figure 2). Using this mask, the PGP 9.5 positive nerve fibres were counted. Further, this mask was used to calculate the area (mm^2^) of PG P9.5 positive nerve fibres associated with myocytes and PGP 9.5 positive nerve fibres within connective tissue. Finally, this mask was also used to detect colocalisation of PGP 9.5 positive nerve fibres with the different peptides of interest, namely SP, NR2B and NGF.

**Table 1:** Presentation of the number of included participants, the number of participants where the obtained biopsies were possible to use for the analysis and the number of participants with only myocytes, connective tissue or both tissues together in their samples

	All recruited participants	Samples with			
		At least one of the tissues meant for analysis	Myocytes only	Connective tissue only	Both tissues together
Number of participants					
All	60	57	26	54	42
Men	30	27	16	24	22
Women	30	30	10	30	20

Haematoxylin (HTX) staining was performed prior to the immunohistochemical analysis on a few sections to assist in the characterisation of cell types within the biopsies. After drying the glass slides in a dark room at room temperature for 24 hours, they were hydrated in a tap‐water bath for 2 min, followed by a 20 min immersion in Harris HTX‐solution (Thermo Fisher Scientific, Göteborg, Sweden). This was followed by dipping them in a solution of 70% ethanol and 0.1% hydrogen chloride, and, lastly, rinsing them in a tap‐water bath for an additional 2 min. The glass slides were then dehydrated in baths of 70% and 95% ethanol, for 2 min each, and immersed in Technical Xylene (Avantor LLC, Radnor, PA USA) for 10 min. Finally, thin glass coverslips were mounted on each slide with Histomount (Thermo Fisher Scientific, Göteborg, Sweden), and micrographs were captured using a light microscope. In skeletal muscle sections, myocytes appear as striated, multi‐nucleated cells which are round or cylindrical in shape.^25^ These myocytes, which are also referred to as muscle fibres or muscle cells, are surrounded by loose connective tissue known as the endomysium that often contains many cells and a loose arrangement of fibres. The connective tissue layer covering bundles of muscle fibres is known as perimysium and contains most of the nerve fibres and blood vessels.^1^ In the present study, the investigated tissues were differentiated based on these histological features.^1^, ^25^ By using this approach, nerve fibres were separated into those associated with myocytes or within connective tissue (Figure 2).^24^ Any fluorescent signal that exceeded the mean background of the picture + 2 SDs (an estimate of the 95% confidence interval), with a minimum length of 4 μm and width of 4 μm, was defined as a positive nerve fibre.^16^ Fluorescent signals that were separated by 5 µm or less, sharing the same path and tissue, were considered as belonging to the same fibre. Finally, SP was used as a marker to identify sensory afferent fibres.

Since most of the images examined contained different amounts of myocytes and connective tissue, the density of innervation in each tissue was calculated separately to avoid bias. This was done by normalising the PGP 9.5 positive counts to the area (mm^2^) of the tissue present in the images, that is number of positive PGP 9.5 fibres in the tissue divided by the total area of the same tissue on the image, and finally, this was averaged over the number of images for each participant. To detect if NR2B, SP and NGF were colocalised with PGP 9.5 positive fibres, the masks for each marker were overlapped using a specific plugin within the ImageJ program known as image calculator. Overlapping signals were counted, and the frequency of expression was calculated using the equation number of PGP 9.5 positive fibres that were colocalised with other substances divided by the total number of PGP 9.5 positive fibres in the image, averaged over the number of images for each participant.

### 2.6 Statistical analysis

For all data and statistical analyses, SigmaPlot for Windows, version 11 (Systat Software Inc, Chicago, IL, USA), was used. The average percentage of PGP.9.5 positive fibres in different tissues was calculated by dividing the average number of positive fibres in either connective tissue or myocytes to the average total number of positive fibres multiply by 100. The data regarding the participants' age and the number of PGP9.5 immunoreactive fibres are presented as mean (±SD). The data regarding the frequency expression of SP, NR2B and NGF alone or in different combinations (ie SP/NR2B; SP/NGF; NR2B/NGF; SP/NR2B/NGF) and the density of fibres were not normally distributed and are therefore presented as median and interquartile range (IQR). The Mann‐Whitney U test was used to test for significant differences between connective tissue and myocytes, and between sexes. Spearman correlation was used to analyse the expression of molecules to the age of participants. For all tests, the level of significance was set to *P *< .05, except for the Spearman correlation test. The level of significance was corrected according to the method of Bonferroni, and since there were seven tests, the p‐value was therefore set to *P *< .007.

## 3 RESULTS

Sixty healthy and pain‐free participants were included, and biopsies were obtained from all without causing any reported side effects. Biopsies obtained from three of the participants did not contain any muscle tissue, so they were excluded from the analysis. An average of 25.3 (± 7.6) PGP 9.5 immunoreactive fibres (positive fibres) was identified per subject, from the biopsy sections of 27 men and 30 women. Most PGP 9.5 positive fibres (72.6%) were found within the connective tissue, while 27.4% were found associated with myocytes.

### 3.1 Differences between connective tissue and myocytes in the density and expression frequency of nerve fibres in the masseter muscle

The density of nerve fibres was significantly greater within connective tissue than with fibres associated with myocytes (*P *< .001) for the entire group. The same results were found when data from men and women were analysed separately (*P *< .001). The frequency of PGP 9.5 fibres expressing NR2B and NGF alone or in combination was significantly greater when fibres were associated with myocytes than within connective tissue (*P *< .001), while the frequency of PGP 9.5 fibres expressing SP alone or in combination with NR2B was significantly greater within connective tissue (*P *< .001). There was no significant difference in the expression frequency of SP/NGF and SP/NR2B/NGF between tissues (Table 2).

**Table 2:** Table presenting the median (IQR) nerve fibre density (fibres/area in mm^2^) as well as the median (IQR) frequency of PGP 9.5 positive fibres expressing SP, NR2B and NGF alone or in combination. Data are presented both combined from myocytes and connective tissue and for each tissue separately. Data from the masseter muscle of 57 healthy and age‐matched participants; 27 men and 30 women

	Density of PGP9.5	The frequency of PGP9.5 fibres expressing different substances alone or in combination						
		SP	NR2B	NGF	SP/NR2B	SP/NGF	NR2B/NGF	SP/NR2B/NGF
Data combined								
All	348.4 (245.9)	48.3% (40.8)	60.3% (49.7)	29.5% (66.1)	22.4% (29.6)	8.9% (14.4)	23.8% (66.5)	8.0% (11.6)
Men	290.4 (235.2)	36.4% (47.8)	63.6% (52.5)	42.8% (68.5)	19.8% (22.8)	7.8% (11.8)	28.6% (70.1)	5.6% (11.2)
Women	376.2 (261.1)	52.9% (36.6)*	58.0% (45.4)	22.9% (40.2)	27.2% (26.5)*	12.0% (13.4)*	20.6% (42.9)	10.1% (14.4)*
Myocytes								
All participants (n = 26)	227.6 (136.1)	11.4% (18.0)	91.4% (11.0)^a^	83.3% (16.7)^a^	8.8% (18.6)	8.9% (18.4)	81.0% (19.1)^a^	8.1% (21.3)
Men (n = 16)	227.6 (137.1)	8.1% (18.9)	91.9% (11.0)^a^	83.3% (12.7)^a^	6.3% (17.9)	6.0% (18.6)	81.0% (12.6)^a^	5.5% (18.6)
Women (n = 10)	266.5 (171.8)	18.0% (19.9)	91.4% (13.7)^a^	85.8% (41.8)^a^	17.2% (19.4)	18.1% (16.4)	81.6% (35.0)^a^	17.0% (19.4)
Connective tissue								
All participants (n = 54)	404.5 (241.2)^a^	57.6% (22.9)^a^	44.9% (26.3)	17.1% (20.2)	29.1% (24.7)^a^	9.2% (10.7)	13.8% (16.5)	8.0% (10.3)
Men (n = 24)	398.4 (209.2)^a^	52.4% (19.3)^a^	41.3% (27.8)	16.7% (23.5)	22.6% (21.5)^a^	8.4% (11.8)	11.6% (18.3)	5.6% (11.0)
Women (n = 29)	415.5 (268.5)^a^	60.0% (26.2)^a^	50.7% (25.5)	19.5% (20.8)	35.9% (27.7)^a^	10.4% (10.9)	14.2% (16.3)	8.7% (10.3)

Note

n = number of participants included in the analysis; IQR = Interquartile range (75 percentile minus 25 percentile).

^a^ Significant differences between myocytes and connective tissue (Mann‐Whitney test; *P *< .05).

* Significant differences between men and women (Mann‐Whitney test; *P *< .05).

### 3.2 Sex differences

**Figure 3:**
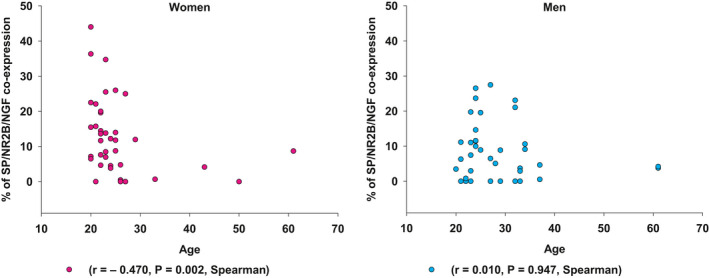
The scatter plots show the correlation between the age of men and women participants and the expression frequency of SP/NR2B/NGF from the data combined

There were no significant sex‐related differences in the density of PGP 9.5 positive nerve fibres. The frequencies of fibres expressing SP (*P *= .024), SP/NR2B (*P *= .018), SP/NGF (*P *= .039) and SP/NR2B/NGF (*P *= .045) were significantly higher in women than in men when combined data from the tissues were analysed. But when the same expression data were analysed in each tissue separately, there was no significant sex‐related difference, although the expression was higher in women (Table 2). Pooled data from both tissues (connective and myocytes) indicated that the frequency of positive nerve fibres expressing SP/NR2B/NGF in combination was negatively correlated with age in women (r = ‐ 0.470, *P *= .002), but not in men (Figure 3).

## 4 DISCUSSION

This study is the first part of a larger project which aims to identify biomarkers of masseter muscle pain in healthy pain‐free subjects and patients with TMD muscle pain. The main finding of the present study is that most of the nerve fibres expressing SP are located within connective tissue. This suggests that nerve fibres within the connective tissue are more likely to serve a sensory function that includes nociception than nerve fibres associated with myocytes. Approximately half of these sensory fibres also express the NR2B subunit of the NMDA receptor, which suggests that SP and NR2B‐receptors play an important role in mediating pain associated with elevated interstitial glutamate concentrations in the muscle. In contrast, this study indicates that few nerve fibres expressing SP are associated with myocytes, which suggests that many of the PGP 9.5 positive nerve fibres associated with myocytes on the other hand are somatic motor efferents. These putative motor efferents displayed a high expression of both NGF and NMDA receptors in this study, the latter could possibly be a reflection of the involvement of NMDA receptors in synaptic transmission to motor neurons centrally.^26^ The elevated levels of NGF in these putative motor axons suggests that they are an important source of exogenous NGF in the masseter muscle. This exogenous NGF could then, in turn, be involved in maintaining the viability of sensory nerve fibres.^11^ Further, this study could also show that there are sex‐related differences in the expression of sensory fibres in the human masseter muscle. Specifically, women displayed a greater expression of sensory afferent fibres (ie SP alone and a combination with NR2B and NGF) than men. This finding could be one explanation for previous reports of greater glutamate‐induced masseter muscle pain intensity in healthy women.^20^, ^21^, ^27^, ^28^ Finally, this study revealed a significant negative correlation of age with the expression of SP, NGF and NMDA receptors in women when data from nerve fibres within connective tissue as well as associated with myocytes were combined. This finding could suggest that the decline in this subtype of sensory afferent fibre in the masseter muscle with age may, in part, explain why the prevalence of women reporting masseter muscle pain declines with age.^29^, ^30^, ^31^

In humans, the NR1 subunit of the NMDA receptor has been shown to be colocalised with glutamate in painful tendons.^32^, ^33^ Certain NR2 subunits of the NMDA receptor, for example the NR2B and D subunits, have been found in the skin of both healthy participants and patients with fibromyalgia.^34^ Since the present study examined expression in different masseter muscle tissues, it is more difficult to compare the outcome with previous studies (human or animal). However, when data from the present study were analysed, without differentiating connective tissue from myocytes, the majority of nerve fibres expressed NR2B and SP (60% and 48%, respectively). Although previous studies have shown that nerve fibres in human masseter muscle have a similar expression of SP and NR2B as rats, the present study showed a slightly greater expression.^16^ One explanation for the greater expression in this study might be the number of human participants in the present study. Given the current finding of differential expression of nerve fibres in the masseter muscle, to improve the internal validity of future studies looking at the expression of biomarkers, it is suggested that expression within connective tissues or associated with myocytes be examined separately.^24^

NGF is essential for the maintenance and survival of sensory and motor neurons.^35^, ^36^ Through the high affinity TrkA receptor, NGF secreted from cells is taken up by small sensory nerve fibres, then transported retrogradely to the cell body.^37^ This process triggers changes in the synthesis of neuropeptides and receptors, which is reflected by altered expression in peripheral and central terminals.^38^ Previous studies have proposed and showed colocalisation of SP and glutamate in small sensory neurons at the level of the dorsal root ganglion (DRG).^39^, ^40^, ^41^ Moreover, it is known that 50% of all neurons expressing SP are C‐fibres and many C‐fibres (73%) are nociceptors.^42^ This indicates that afferent fibres containing SP are most likely nociceptors. The current study showed that nerve fibres in the masseter muscle had a higher expression of NGF when associated with myocytes when compared with those in connective tissue. On the other hand, the current study found a higher expression of SP on PGP 9.5 positive nerve fibres within the connective tissue than those associated with myocytes. These findings together allow for speculation that the vast majority of nerve fibres associated with myocytes are motor axons, whereas those within connective tissue are likely to be sensory afferent fibres.

In rats, glutamate excites masseter muscle afferent fibres through the activation of peripheral NMDA receptors. The magnitude of this glutamate‐evoked afferent discharge is greater in females than males.^20^, ^27^, ^43^ The frequency of expression of NMDA receptors in masseter ganglion neurons was greater in female than in male rats and this difference appears to be due to an oestrogen‐mediated increase in expression of peripheral NMDA receptors.^22^ Three days after injection of NGF into the masseter muscle, rat masseter ganglion neurons expressing NR2B showed a higher expression of CGRP and SP in female than in male rats.^16^ The present study also found that nerve fibres expressing SP alone and in combination with NR2B and NGF are more common in women than in men. Moreover, the same, though non‐significant difference in expression between sexes, was detected when data from both myocytes and connective tissue were analysed separately (Table 2). These findings together suggest that sex‐related differences in experimentally induced muscle pain are due to a higher expression of NR2B and NGF by masseter muscle afferent fibres in women than in men.

Ageing is associated with different neurobiological changes in the peripheral nervous system that might impact the processes of nociception. In aged rats, a loss of thermal sensitivity was associated with a reduction in the expression of voltage‐gated sodium channels (Nav1.8) and transient receptor potential vanilloid 1 (TRPV1) channels in the DRG and skin peripheral nerve fibres.^44^ Other rat studies have reported a decline in the expression of NGF receptors (TrkA and p75) as well as in the axonal transport of CGRP in the DRG with advancing age,^45^, ^46^ which may lead to a reduction in the effect of NGF on sensory nerve fibres by ageing. Moreover, it has been shown that ageing in mice reduces the number and density of both myelinated and unmyelinated tibial peripheral nerve fibres.^47^ A greater reduction in unmyelinated peripheral nerve fibres in both human and different animal studies by ageing has also been demonstrated.^5^ The present study supports these findings from previous studies by presenting a lower co‐expression of neuropeptides (in this case SP) with other biomarkers (NGF and NR2B) in peripheral nerve endings of the masseter muscle of older women. This may be one factor underlying the decreased prevalence of masticatory muscle pain complaints as women age.

### 4.1 Study limitations

Due to methodological limitations, not all biopsies obtained from participants contain myocytes and/or connective tissue. Although a guiding needle was used to assure that the biopsy was obtained from the same area of the muscle, one cannot be certain that exactly the same compartments of the masseter muscle were obtained. This could be considered a limitation, since the different compartments could have different innervation. Another limitation is that the study set‐up did not allow for examination of nerve fibre diameter. For example, it cannot be determined what proportion of nerve fibres associated with myocytes were motor axons as opposed to spindle or other sensory afferent fibres. In order to minimise the possibility of overestimating the expression of sensory fibres, SP was used as a marker to differentiate sensory from motor axons. On the other hand, since SP is not expressed by many sensory afferent fibres, this could instead underestimate the number of afferent fibres in the muscle. While nerve fibres were classified as associated with either myocytes or within connective tissue, the method employed cannot differentiate between nerve fibre endings and nerve fibres that simply passed through the tissue. Thus, it cannot be stated definitively what tissue the individual nerve fibres examined actually innervated. Finally, most of the participants in the study were young, with only a small number of older participants. This could explain why some assessments of age‐related changes in expression did not reach significance.

## 5 CONCLUSIONS

The current study showed that nerve fibres expressing SP, NR2B and NGF are differentially distributed within the tissues of healthy human masseter muscle. The majority of nerve fibres expressing SP, which was used to identify sensory afferent fibres including those involved in nociception, were found within connective tissue. Moreover, there was a greater frequency of sensory afferent fibres that expressed NR2B and NGF in the masseter muscle of women than of men. This finding supports previously proposed theories that have suggested the importance of the expression of these markers in explaining sex‐related differences in masseter muscle nociception. Further, the negative correlation between age and sensory nerve fibres, that is putative nociceptors, may explain why sex‐related differences in muscle pain are more noticeable in younger as compared with older women.

## 6 COMPETING INTERESTS

The authors declare that they have no competing interests.

## AUTHORS’ CONTRIBUTION

Abdelrahman M. Alhilou (AA): Performed the research, analysed the data and drafted the manuscript. Akiko Shimada (AS): Collected the research data, participated in the research design and manuscript editing. Camilla Svensson (CS): Participated in data analysis and manuscript editing. Malin Ernberg (ME): Participated in the research design, data analysis and manuscript editing. Brian E. Cairns (BC): Participated in the research design, data analysis and manuscript editing. Nikolaos Christidis (NC): Designed the research, drafted the manuscript and participated in data analysis.

### Peer Review

The peer review history for this article is available at https://publons.com/publon/10.1111/joor.13109.
